# Biopriming of seed with plant growth-promoting bacteria for improved germination and seedling growth

**DOI:** 10.3389/fmicb.2023.1142966

**Published:** 2023-02-28

**Authors:** Angelika Fiodor, Nur Ajijah, Lukasz Dziewit, Kumar Pranaw

**Affiliations:** Department of Environmental Microbiology and Biotechnology, Institute of Microbiology, Faculty of Biology, University of Warsaw, Warsaw, Poland

**Keywords:** plant growth-promoting bacteria, phosphate solubilization, abiotic stresses, potassium solubilization, auxin, biopriming, seed germination

## Abstract

Several seed priming methods can be used to improve seed germination, seedling vigor, and to overcome abiotic stress. In addition to these benefits, only the biopriming method provides the additional benefit of biotic stress management, earning it special attention. Seed biopriming is useful in almost all crops around the world and is an environmentally friendly alternative to chemical fungicides. Biopriming usually refers to use of beneficial microorganisms, in particular plant growth-promoting bacteria (PGPB) able to survive under various harsh environmental conditions. In this study, various bacterial strains were isolated from samples of different origins, i.e., rhizospheric soil, desert sand, and sea mud. Preliminary screening of 156 bacterial isolates was conducted on the basis of their potassium (K), phosphorus (P) solubilization ability, and production of plant growth hormone, i.e., indole acetic acid (IAA). The most efficient bacteria were identified by 16S rRNA gene nucleotide sequences and further examined for their ACC deaminase activity, ammonia production, and biocontrol activity (defined *via* chitinolytic activity, HCN, and siderophores production). Finally, carrot seed germination assay was conducted with 10 shortlisted most potent isolates. 68.6, 58.3, and 66.7% of tested bacterial isolates were capable of P, K, and Zn solubilization, respectively. *Klebsiella aerogenes* AF3II1 showed the highest P and K solubilization, while isolate AF4II5, AF7II3, and PC3 showed the highest IAA synthesis ability. *Serratia plymuthica* EDC15 and *Pseudomonas putida* AF1I1 showed the strongest chitinolytic and siderophore production activity, respectively. Seven isolates demonstrated strong HCN production ability. Five isolates improved carrot seed germination. Only selected isolates with plant growth-promoting properties can improve carrot germination. The results of this study demonstrate that mainly auxins are involved in seed germination. Furthermore, the data suggest that phosphate solubilization ability may play an additional role in seed germination.

## Introduction

Seed germination is a key developmental transition in plant life and therefore extremely essential for plant cultivation (Zhao et al., 2021). During this process, many biochemical changes occur due to a constantly operating network of genes and hormones. The speed, uniformity, and quality of seed germination significantly impact the plant’s subsequent growth and condition. Seed biopriming has attracted much attention for its potential to induce seed germination, and provide early support for seedling growth under stress conditions. Seed biopriming techniques include biological seed germination enhancement using various microorganisms and microbe-mediated signaling molecules, with seed coating, pelleting, hardening, etc. (Prasad et al., 2016). Natural microbial communities associated with outer space of the seed can enter to the seed. Increase of the effectiveness of its hydration shortens the imbibition time, which consequently lift the germination rate (Mahmood et al., 2016). It was already proved that the use of microbial inoculants with plant growth-promoting bacteria (PGPB) can increase crop production by 12–20% and biopriming of seeds with various beneficial bacteria, especially PGPB, increases the plant resilience and effectiveness under adverse conditions (Pérez-Jaramillo et al., 2016).

Soil is considered the most complex ecosystem and is the scene of numerous interactions between organisms. Soil bacteria able to colonize rhizosphere appear to play a key role in plant disease prevention and nutrient supply. For this reason, microbial fertilizers containing PGPB are an exceedingly attractive alternative to the traditional chemical approach. The introduction of bioformulations containing beneficial bacteria into cultivation aims to maintain long-term soil fertility and ensure crop productivity (Chen et al., 2021). This offers innumerable benefits and is in line with sustainable agriculture (Saleem et al., 2021). Many studies have demonstrated the positive effect of PGPB application on plant growth and yield (Pandey and Gupta, 2020; Fahsi et al., 2021). Beneficial bacteria can boost plant growth and vigor through direct and indirect mechanisms of action (Saleemi et al., 2017). Direct mechanisms can be described as those that act on plants by secreting plant growth regulators such as phytohormones or by enhancing nutrient supply from natural resources (Backer et al., 2018; Basu et al., 2021). Selected bacteria may transform insoluble inorganic nutrient and their organic forms into soluble forms available for plant uptake through solubilization or mineralization, respectively. Strains that have the ability to solubilize zinc from zinc oxide and other sources have been shown to benefit various crops such as cotton and wheat (Kamran et al., 2017; Ahmad et al., 2021). Many biopriming agents or biostimulants also upgrade plant resilience by stimulating root and shoot growth. This allows plants to explore deeper soil layers during the drought season, stimulate synthesis of compatible solutes to restore favorable water potential gradients, and increase water uptake as soil water levels fall (Van Oosten et al., 2017). Moreover, indirect action by certain bacteria may enhance plant growth through pathogen suppression or increasing abiotic stress tolerance (Grover et al., 2021). PGPB can improve plant tolerance to abiotic stress by inducing favorable alteration in physical, chemical, and biological responses (Fatima et al., 2020; Fatima and Arora, 2021; Grover et al., 2021).

The evolving market for biofertilizers in response to climate change and the transition to sustainable agriculture requires a constant search for new microorganisms. Meeting certain expectations is associated with the selection of microorganisms with desired properties, preferably with a broad spectrum of activity (Basu et al., 2021). In addition, the multiplicity of environmental factors together with the presence of pathogens causing abiotic stress is a challenge in these times (de la Torre-Hernández et al., 2020; Garcia Teijeiro et al., 2020).

In this study, we aimed to define a strategic screening method for PGPB, including isolation from the samples collected in different environments, to investigate their potential applications in biopriming seeds (carrot, i.e., *Daucus carota* sp. sativus) for sustainable agricultural production. Another objective was to select beneficial bacteria with biostimulatory potential for seed germination and attempt to link them to plant growth-promoting biocontrol properties.

## Materials and methods

### Soil sampling and isolation of bacteria

In this study, 12 rhizospheric soil samples, four sand samples from the Błędowska Desert, one sample from Dead Sea mud in Israel, and six vegetables root samples were used for isolation of potential PGPB isolates. Rhizospheric samples of cereal and vegetable crops (*viz.* rye, wheat, maize, parsley, beet root, and carrot) were collected from different cities of northeastern Poland. Samples were collected in sterile plastic bags and stored at 4°C before isolation of microorganisms. One gram of soil from each sample was inoculated into LB medium at 30 ± 1°C for 5 days, then serially diluted and spread onto LB agar medium plates. After incubation, morphologically distinguishable (e.g., shape and color) colonies were transferred to fresh LB agar medium plates. Isolated bacteria were purified and stored in LB media with glycerol at 4°C for further study and at −80°C for collection. The endophytic isolates were obtained by isolation from the inner part of the vegetable root according to the method of Cochard et al. (2022) with some modifications. A detailed information on the isolates and their sample source in this study can be found in Supplementary Table S1.

### Screening strategy for selection of most potent PGPB strains

The overall screening strategy was divided into two stages. The primary and secondary screening were based on different qualitative and quantitative estimation of different plant growth-promoting (PGP) attributes and biocontrol potential.

### Primary screening of bacterial isolates

The primary screening was carried out on the basis of phosphate and potassium solubilization and auxin production. For the screening experiments, each isolate was initially inoculated in LB broth and incubated at 30 ± 1°C under agitating conditions (160 rpm) for 48 h [until the optical density (OD660) reached 0.8 to 1.2] to obtain a fresh liquid culture of bacteria. The screening experiments were performed with three replicates.

#### Qualitative estimation of phosphate (P) and potassium (K) solubilization

The qualitative phosphate (P) and potassium (K) solubilization ability of all isolates were evaluated using NBRIP agar medium with tricalcium phosphate (Ca_3_(PO_4_)_2_) as P source and Aleksandrow agar media with feldspars (K_2_O·Al_2_O_3_·6SiO_2_), i.e., an aluminosilicate mineral as a K source (Pranaw et al., 2020; Boubekri et al., 2021). Briefly, 3 μL of 48 h fresh inoculum of each isolate was spotted onto NBRIP agar plate media and incubated at 30 ± 1°C for 5 days. The potential for P solubilization of the specific isolate was demonstrated by the existence of a halo zone surrounding the sites of bacterial growth. The phosphate solubilization index (PSI) and potassium solubilization index (KSI) were determined by calculating with the following formula (Boubekri et al., 2021):


Eq. 1 PSI/KSI/ZSI/SPI = diameter of the clearing zone    +colony diametercolony diameter

#### Quantitative estimation of auxin, i.e., indole-3-acetic acid (IAA) production

IAA production was detected by the colorimetric method as described by Hartmann et al. (1983) with some modifications adapted by Pranaw et al. (2020). Briefly, 2% (v/v) of bacterial liquid culture was inoculated into Jensen’s broth supplemented with 5 mM tryptophan and incubated for 5 days at 30 ± 1°C and 200 rpm. Samples were collected after 48 and 120 h of incubation, and centrifuged at 10,000 rpm for 10 min. Then, the Salkowski reagent (120 mL 95% sulfuric acid and 2 mL 0.5 M FeCl_3_, 200 mL ddH_2_O) was added to the supernatant in a ratio of 4:1 and left in the dark for 30 min. IAA was quantified using a spectrophotometer at 530 nm. The calibration curve was prepared using commercial pure IAA (Sigma Aldrich, St. Louis, MO, United States) in 50% ethanol solution at concentration range of 1 to 1,000 μg mL^−1^.

### Secondary screening of shortlisted bacterial isolates

The primarily shortlisted isolates were further examined for their other PGP attributes, which includes qualitative estimation of zinc solubilization, and ACC (1-aminocyclopropane-1-carboxylate) deaminase production, and quantitative estimation of ammonia production. Biocontrol potential such as siderophore and hydrogen cyanide (HCN) synthesis and chitinolytic activity were also included in the secondary screening.

#### Zinc solubilization

Zinc (Zn) solubilizing ability of bacterial isolates were screened using a basal medium supplemented with Zn (Dinesh et al., 2018). Medium was augmented with insoluble Zn, separately from zinc oxide (ZnO, 0.15%), zinc carbonate hydroxide basic ([ZnCO_3_]_2_ [Zn(OH)_2_]_3_ 0.15%), and zinc carbonate (ZnCO_3_ 0.1%). The volume of 3 μL of fresh culture of each isolate was spot inoculated onto the Zn-supplemented media plates and incubated at 30 ± 1°C for 4 days. Zn-solubilizing isolates produced clear zones around colonies. The appearance of a clarification zone around the colonies indicated positive solubilization. The diameter of these zones was recorded. The Zn solubilization index (ZSI) was determined by using Equation 1.

#### ACC deaminase production

The ACC deaminase activity of isolates was quantitatively analyzed based on their ability to assimilate ACC as a sole nitrogen source according to Pranaw et al. (2020). Nitrogen-free DF (Dworkin and Foster) salt medium with ACC-supplemented plates was spot inoculated and incubated 30 ± 1°C for 5 days. In the control plates, ammonium sulfate was used as the sole nitrogen source. Growth of the isolates on ACC-supplemented plates proved the deaminase activity of the enzyme. Their growth on control plates corroborated the negative results.

#### Chitinolytic activity

The chitinolytic properties of all shortlisted isolates were screened qualitatively on the chitinase-detection agar (CHDA), according to Drewnowska et al. (2020). Colloidal chitin was prepared and stored at 4°C until use (Drewnowska et al., 2020). Briefly, 3 μL of fresh culture of each isolate was spot inoculated onto CHDA plate medium and incubated at 30 ± 1°C for 10 days. Bacteria with break-down chitin ability were determined after 5 and 10 days at 30 ± 1°C by visualizing the clear zone around the colonies. Chitinolytic index (CHI) was also determined by using the Equation 1.

#### Siderophores production

Siderophore production potential were quantified using the modified chromazurol S (CAS) agar methodology described by Pranaw et al. (2020). 3 μL of freshly grown cultures was spot inoculated using on nutrient agar (NA) medium supplemented with CAS dye solution and incubated for 10 days. A yellow-orange halo zone formation CAS agar around the colony confirmed siderophore production. The diameter of these zones was recorded. Further, the siderophores production index (SPI) was also determined using Equation 1.

#### Hydrogen cyanide (HCN) production

HCN production was analyzed according to the methodology described by Pranaw et al. (2020). Freshly grown isolates were streaked over KB agar medium plates amended with 4.4 g L^−1^ glycine. A single sheet of filter paper (size 90 mm) was soaked with 0.5% picric acid solution in 2% sodium carbonate, and placed in the lid of each plates. All Petri plates were sealed with parafilm and incubated at 30 ± 1°C for 5 days. A positive result was checked by color changed in the filter paper from yellow to orange-brown tone. The rich yellow hue of the filter paper remaining unaltered following the development of bacteria indicates a negative test. Results were compared to control and categorized as negative (−) for no color change, moderately positive (++) for orange color, and highly positive (+++) for red-brown color. Each assay was repeated at least twice, and all assays were done in triplicate.

#### Quantitative estimation of ammonia production

Quantitative estimation of ammonia production was performed for all shortlisted isolates according to Mukherjee et al. (2019) with some modifications. One milliliter of the 48 h grown culture of each isolate with an optical density (OD_600_ ~ 0.8–1.2) was inoculated into 40 mL of peptone broth medium and incubated at 30 ± 1°C. Samples were collected after 1, 3, 5, and 7 days of incubation. 40 μL of K-Na tartrate (0.177 M) and 40 μL of Nessler reagent were added to 2 mL of sample supernatant. The reaction mixtures were mixed thoroughly using vortex and kept in dark for 10 min. Color change of the solution from yellow to orange/dark brown after 10 min of incubation indicated ammonia presence. The OD was measured at 425 nm and ammonia concentration was calculated using standard curve of ammonium sulfate ((NH_4_)_2_SO_4_) at 0.6–10 ppm under standard reaction condition.

### *In vitro* compatibility test among microbes

Compatibility was checked for selected bacterial isolates using agar diffusion test according Santiago et al. with some modification (Santiago et al., 2017). Freshly grown cultures were streaked vertically onto freshly prepared LB agar medium. Next, the second isolate was streaked outward at an angle of approximately 90° from the resulting colonies of the first isolate and incubated at 30 ± 1°C. After 72 h, the colony lines were observed and the presence of inhibition zones at the crossing points of the paired isolates was noted.

### Identification and biochemical characterization of selected PGPB isolates

For Molecular identification, the genomic DNA of selected PGPB strains were isolated using Bacterial & Yeast Genomic DNA Purification Kit (EURx) following the manufacturer’s instructions. Bacterial DNA was quantified using the NanoDrop 2000 spectrophotometer (Thermo Fisher Scientific, Wilmington, United States) and by gel electrophoresis in ChemiDoc XRS+ (Bio-Rad, United States). Near full-length 16S rRNA gene sequences were amplified by polymerase chain reaction (PCR) in Eppendorf AG thermal cycler (Eppendorf, Hamburg, Germany) utilizing pair of primers: 27F (AGAGTTTGATCCTGGCTCAG) and 1492R (GGTTACCTTGTTACGACTT) (Haque et al., 2020). Amplification was done under following conditions: 5 min at 94°C, 30 s at 94°C (×30), 45 s at 57°C, elongation for 1.5 min at 72°C, and a final extension for 10 min at 72°C. The DNA quality was determined using NanoDrop 2000 Spectrophotometer and gel electrophoresis. The amplified products were purified using the PCR purification kit (EPPiC Fast reagent kit, A&A Biotechnology, Poland) following the manufacturer’s protocol. The purified PCR products were further sequenced using different 16S rRNA gene sequencing primers: 341 F (5′CCTACGGGNGGCWGCAG3′); 928F (5′TAAAACTYAAAKGAATTGACGGG3′); and 518 R (5′ATTACCGCGGCTGCTGG3′) at Eurofins Genomics, Germany GmbH. The gene sequences were further assembled with the help of BioEdit Sequence Alignment Editor v7.0.5.3 software. To identify the closest taxa, the 16S rRNA gene sequences were compared to sequences from the National Center for Biotechnology Information (NCBI; United States)^1^ nucleotide nr/nt (non-redundant nucleotide) database using the BLAST algorithm. The phylogenetic analysis and construction of the phylogenetic tree was performed using MEGA7 for 33 isolates with Neighbor-Joining (NJ) algorithm. The branch quality of all generated trees was evaluated using 1,000 bootstrap replicates. The biochemical profiling of selected PGPB isolates was characterized using HiCarbo kit KB009 (Hi-Media Laboratories, Mumbai, India) following the manufacturer protocol (Pranaw et al., 2020).

### Abiotic stress tolerance of selected strains

Using the appropriate medium, 10 shortlisted PGPB isolates were tested for their ability to withstand various abiotic stresses, including salt, pH, and osmotic stress (drought). After incubation, the development of isolates under various abiotic stress scenarios was compared to that of isolates produced on typical nutrient agar plates at 28 ± 1°C. Results were compared to control plates/flask and categorized as negative (−) for no growth, moderately positive (++) for growth that was more but still less than the control plates/flask, and highly positive (+++) for growth that was comparable to the control plates/flask. Each assay was repeated at least twice, and all assays were done in triplicate.

#### Salinity tolerance

To study the salt-tolerant behavior of bacterial isolates, all isolates were inoculated on LB agar medium supplemented with different NaCl concentrations (ranging from 0 to 15%) (Pranaw et al., 2020).The growth of colonies on the plate was recorded by the naked eye after 5 days of incubation at 30 ± 1°C.

#### pH tolerance

All isolates were tested for their tolerance to grow under various pH conditions on solid media using LB medium with different pH values (3.0, 3.5, 4.0, 5.0, 6.0, 8.0, and 10.0). LB media was adjusted to desired pH with NaOH and HCl. For significantly lower pH medium (i.e., 3.0 and 3.5), LB broth was used, whereas the agar concentration was increased to 20 g/L at pH 4. The inoculated plates and tubes were incubated for 5 days at 30 ± 1°C. OD at 600 nm was used to monitor growth in the broth medium, while plate observation of growing colonies was done with the naked eye (Pranaw et al., 2020).

#### Osmotic stress (drought) tolerance

The effect of drought stress on bacterial growth was determined by growing the PGPB isolates in LB broth containing polyethylene glycol (PEG) 8,000 (Thermo Fisher Scientific Inc., United States) 0, 15.8, 23.9, and 30.7% to obtain different water potentials (0, −0.35, −0.75, and, −1.2 MPa), respectively (Michel, 1983). The calculation of PEG required to obtain different potentials (Ψ) in particular temperatures (T) was performed according to the following equation:


Eq. 2 $$PEG=\left(4-{\left(5.16\Psi T-560\Psi +16\right)}^{0.5}\right)/\left(2.58T-280\right)\;$$

The inoculated flasks were incubated at 30 ± 1°C, 150 rpm for 5 days. The growth was measured *via* spectrophotometer at 600 nm (Ashry et al., 2022).

### Seed germination test

Unprimed carrot seeds were selected for the seed germination assay and collected from the local market, Poland. The seed surface sterilization was performed following the method of Pandey and Gupta (2020) with minor modifications. In brief, seeds were surface sterilized with 2% sodium hypochlorite solution and 70% ethanol for 2 min and 1 min, respectively. The sterilized seeds were soaked in bacterial suspension (10^7^ CFU/mL) for 2 h, while in case of control, the seeds were soaked in ddH_2_O. After soaking, the seeds were removed from the liquid and placed over autoclaved Whatman filter paper inside Petri plate. Before seeds placement, the filter paper was moistened with 5 mL of bacterial suspension, sterilized ddH_2_O. Each Petri plate contained 15 seeds in 3 replicates. The plates containing carrot seeds were placed in a growth room with a daily temperature of 20 ± 1°C and incubated for 6 days, respectively. After incubation, the seeds were monitored and the germination index or germination percentage was evaluated using Equation 3 (Fahsi et al., 2021).


Eq. 3 $$\mathrm{Germination Index}=\frac{\mathrm{number of germinated seeds}}{\mathrm{Total no of seeds}}\;\times \;100$$

Further, relative seed germination with respect to control is also calculated using following formula:


Eq. 4 Relative seed germination=number of germinated seeds in treatmentnumber of germinatedseeds in control×100

### Statistical analysis

Statistical analyses we performed using R version 1.2.1335 program. One-way analysis of variance was performed to evaluate the effect of bacterial isolates on carrot seed germination. Differences between means at *p* < 0.05 were evaluated by Tukey HSD test.

## Results and discussion

In this study, bacteria were isolated from various environments such as bulk soil, vegetables, desert sand, and sea mud and then subjected to screening for plant growth-promoting traits. Moreover, molecular identification of the most promising plant growth-promoting bacteria was performed. Finally, the potential growth-promoting abilities were evaluated by germination test using carrot seeds.

### Preliminary screening

**Figure 1:**
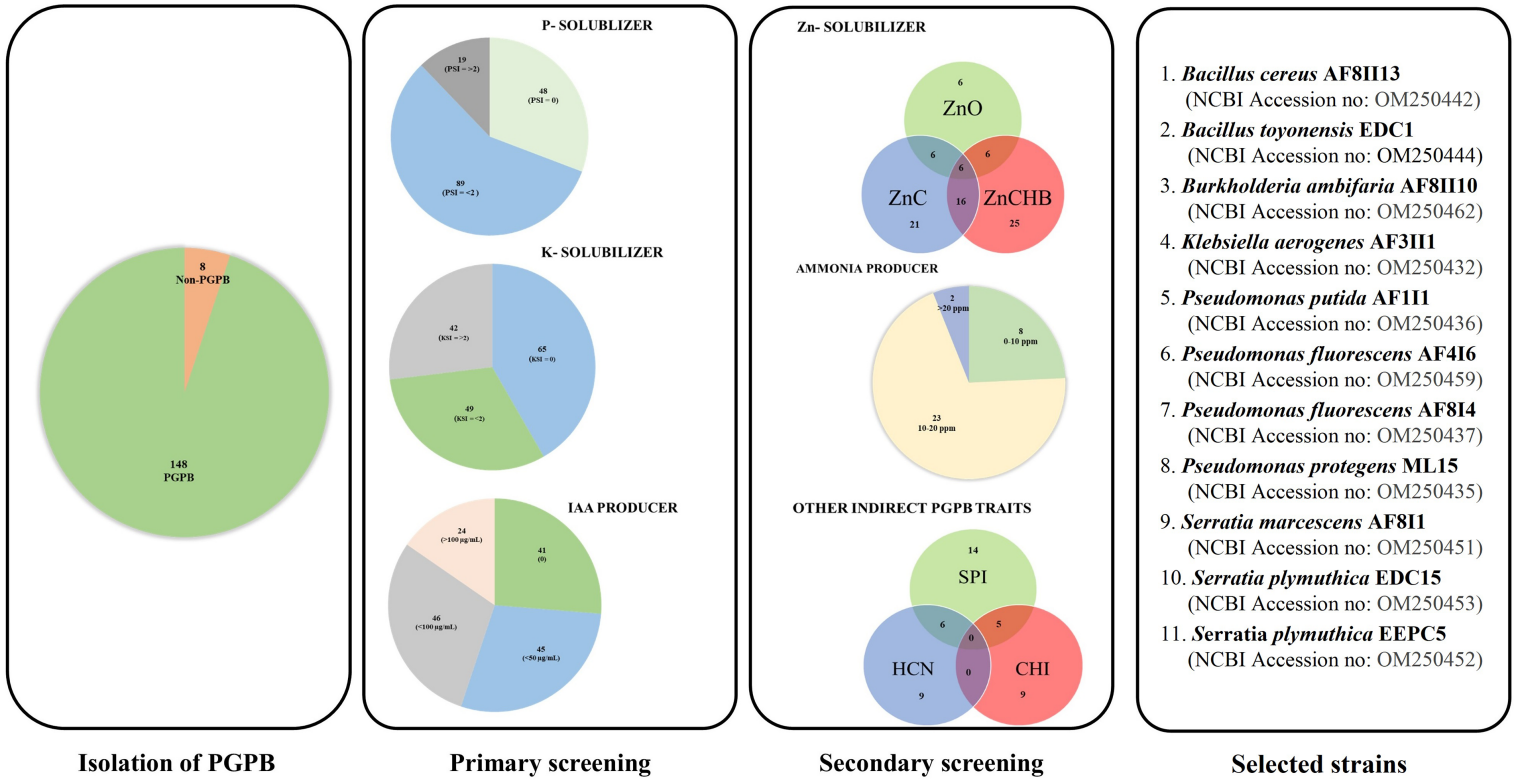
Strategical screening to select the most potential PGPB strain from 156 microbial isolates with a different sample of origin.

One hundred and fifty-six bacterial cultures were evaluated for their ability to solubilize inorganic phosphate and potassium. The isolates obtained were from samples from ecologically very diverse habitats. Among them, 87 strains were isolated from rhizospheric soils of different crops, and 32 and 29 isolates were obtained from samples from the rhizospheric and endophytic parts of vegetables, respectively. Only eight isolates were obtained from environments under abiotic stress conditions. Four and three isolates were obtained from the Błędowska Desert and the Dead Sea, respectively. Nearly 67 and 58% of the isolates showed the ability to solubilize phosphorus and potassium, respectively (Figure 1 and Supplementary Table S1), which is similar to other studies (Setiawati and Mutmainnah, 2016; Ashfaq et al., 2020). Isolate AF3II1 manifested the highest PSI value (3.67), followed by isolates AF1I8 and AF8I4. In the potassium solubilization test, the highest KSI values of 5.25, 3.67, and 3.60 were obtained for isolates AF3II1, AF2II2, and PC5, respectively. High PSI and KSI values were observed for the tested isolates, which is consistent with the results of other studies for other soil bacteria (Shin et al., 2015; Setiawati and Mutmainnah, 2016; Iyer et al., 2017; Sarikhani et al., 2018; Chen et al., 2020; You et al., 2020; Yaghoubi Khanghahi et al., 2021). 17.8 and 46.2% of isolates that exhibited solubilization ability had a value of PSI and KSI, respectively, that was greater than or equal to 2.0. This indicates that some of the strains tested have a high potential to solubilize phosphate and potassium. In addition, half of the isolates showed the ability to solubilize both sources of elements, and the occurrence of these traits is not related. Many of the benefits in agriculture, such as yield increases, have already been described when bacteria with the ability to solubilize phosphate and potassium were used (Liu et al., 2011; Yaghoubi Khanghahi et al., 2019; You et al., 2020).

Auxins production are one of the most important traits of bacteria that promote plant growth. It is estimated that up to 80% of microorganisms in soil communities are capable of synthesizing IAA, although the amount of hormone produced may depend on the microbial isolate (Govind et al., 2015; Maldonado et al., 2020). In the present study, quantification of IAA after 48 and 120 h showed that over 71 and 78%, respectively, of the 156 isolates tested were able to produce this hormone (Supplementary Table S1). The results obtained are in agreement with many data previously presented for different environments (Indah et al., 2017; Ashfaq et al., 2020; Lebrazi et al., 2020). The IAA concentration in the culture medium ranged from 0.12 and 0.26 to 171.18 and 215.29 μg mL^−1^ after 48 and 120 h, respectively, in this assay. Isolates AF4II5, AF7II3, and PC3 had the highest IAA concentration of 171.18, 147.18, and 144.24 μg mL^−1^, respectively (after 48 h of incubation). Further incubation up to 120 h resulted in the detection of IAA production in 11 additional isolates compared to the initial number of IAA producers (after 48 h). Isolates AF6I6, AF7I2, and AF6I5 had the highest IAA concentration after 120 h of incubation, 202.82, 210.12, and 215.29 μg mL^−1^, respectively. Surprisingly, IAA concentration decreased in 27 of the isolates tested in this research after 120 h compared with 48 h. The decrease in IAA content in culture media is consistent with other studies suggesting degradation by microbial oxidase and IAA peroxidase (Raut et al., 2017; Lebrazi et al., 2020).

### Identification of bacteria

**Figure 2:**
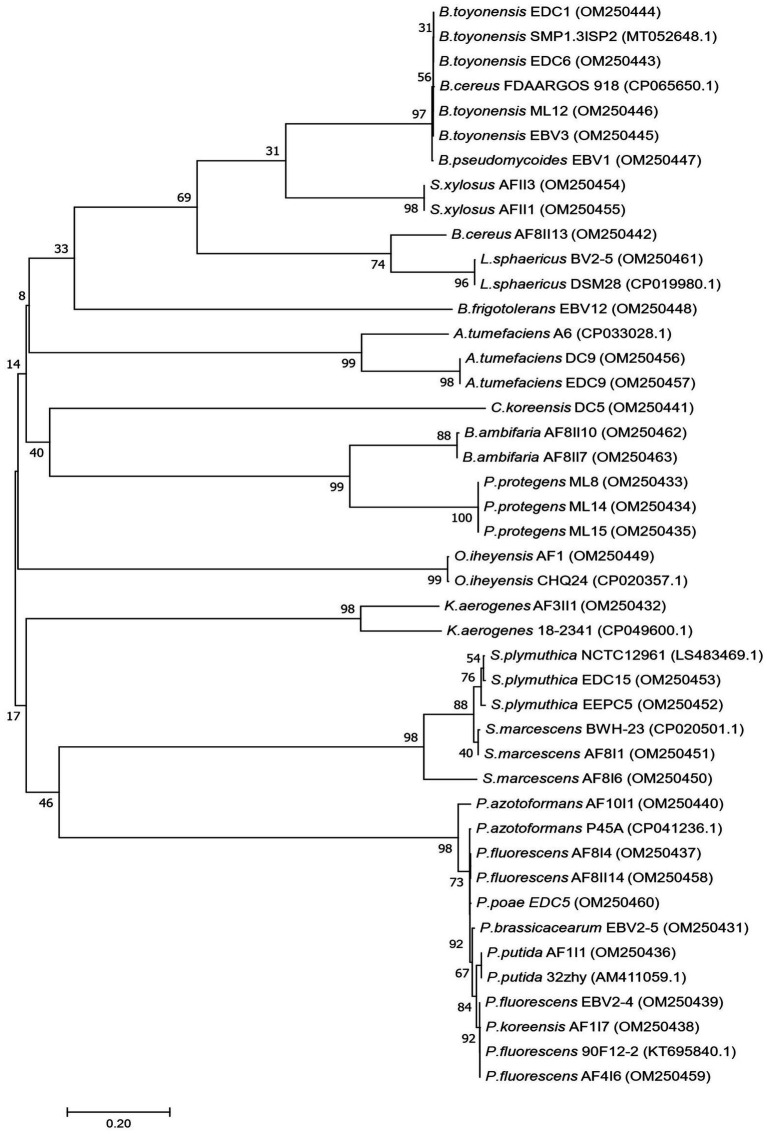
Phylogenetic tree showing the relationship of the 33 newly isolated strains, based on their 16S rRNA gene sequences using the Neighbor-Joining (NJ) algorithm.

Based on primary screening, 33 potentially efficient isolates for plant growth promotion with several studied features were selected. In the next step, they were subjected to molecular identification by partial 16S rRNA gene sequencing of a fragment of length 1,478–1,513 bp. The NCBI GenBank accession number of sequences, closely related species, and similarity value are listed in Supplementary Table S2. Phylogenetic tree was also constructed using fragments of 16S rRNA gene sequences (Figure 2). Comparison of obtained sequences with the deposited sequenced showed that 11 genera of bacteria were present. The largest number, i.e., 12 isolates, was assigned to the genus *Pseudomonas*, among which the most abundant were *Pseudomonas fluorescens* (AF4I6, AF8I4, AF8II14, EBV2-4), *Pseudomonas azotoformans* (AF10I1), *Pseudomonas brassicacearum* (EBV2-5), *Pseudomonas koreensis* (AF1I7), *Pseudomonas poae* (EDC5), *Pseudomonas protegens* (ML8, ML14, ML15), and *Pseudomonas putida* (AF1I1). The bacteria isolated from the extremely saline environment were recognized as *Oceanobacillus iheyensis* (AFI) and *Staphylococcus xylosus* (AFII1, AFII3). The genus *Bacillus* was represented by isolates from soil (*Bacillus cereus* AF8II13), sand samples of the Błędowska Desert (*Bacillus toyonensis* ML12) and from endophytic parts of vegetables, i.e., carrots (*B. toyonensis* EDC1 and EDC6) and beets (*Bacillus pseudomycoides* EBV1 and *B. toyonensis* EBV3). Two isolates of *Agrobacterium tumefaciens* were isolated from the rhizosphere (*A. tumefaciens* DC9) and the endophytic part of the carrot root (*A. tumefaciens* EDC9). One isolate DC5 from carrot samples was identified as *Comamonas koreensis*. *Brevibacterium frigoritolerans* was represented by one isolate *Brevibacterium frigoritolerans* EBV12 from the endophytic part of beet root. Two isolates of *Burkholderia ambifaria* (*B. ambifaria* AF8II7, *B. ambifaria* AF8II10) were obtained from cereal rhizosphere samples. Isolate AF3II1 was classified as *Klebsiella aerogenes* and BV2/5 as *Lysinibacillus sphaericus*. Of the isolates tested, four were identified as *Serratia*, i.e., AF8I1 and AF8I6 are *Serratia marcescens* and EDC15 and EEPC5 are *Serratia plymuthica* DC5.

### Secondary screening of shortlisted bacterial isolates

The selected isolates were further characterized based on Zn solubilization, ACC deaminase, and ammonia production. Implementation of bacteria with Zn solubilization activity allows Zn supplementation in available form in soil when needed (Nagaraju et al., 2020). Although zinc can be artificially incorporated into the soil, similar to P and K, it is immediately and almost completely fixed and immobilized, making it inaccessible to plants (Bhatt and Maheshwari, 2019; Sher et al., 2022).

Zinc solubilization ability has been evaluated for several sources of Zn: zinc oxide, zinc carbonate hydroxide basic, and zinc carbonate in plate test. Solubilization of zinc carbonate hydroxide basic and zinc carbonate was demonstrated by 70% of the isolates tested (Supplementary Table S3). *O. iheyensis* AF1 showed the highest solubilization ability (5.1) for zinc carbonate hydroxide basic, while isolate *K. aerogenes* AF3II1 revealed the highest solubilization potential for zinc carbonate at 3.33. Only 18.2% of the isolates showed solubilization activity for zinc oxide. The two isolates *Serratia marcescens* AF8I1 and AF8I6 showed the highest dissolution for zinc oxide with a solubilization index of 3.7.

The data show that maximum size of halo zones was slightly larger for zinc oxide, followed by ZnCO_3_ and 2ZnCO_3_ × 3Zn(OH)_2_, which is consistent with previous reports (Dinesh et al., 2018). It is worth emphasizing that there were relatively few isolates with solubilization ability of all zinc sources used in analysis, which is comparable to the study by Bhatt and Maheshwari (2019).

The ability to convert nitrogen into a form soluble by plants is another attractive trait sought for potential plant growth-promoting microbes (Sharma et al., 2021). In the current study, all of tested isolates showed the ability to convert organic nitrogen from peptone into ammonia (Supplementary Table S3). The concentrations of ammonia produced differed greatly between isolates and time of incubation from 0.345 to 27.20 ppm. A similar trend was demonstrated in bacterial isolates from the rhizosphere of cotton and chick pea (Ahmad et al., 2008; Iyer et al., 2017). Data showed that isolates of *P. fluorescens*, *B. ambifaria*, *S. xylosus*, and *P. brassicacearum* were characterized by high ammonia production. The highest ammonia production after 24 h of incubation was observed for *S. xylosus* AF1 (13.5 ± 0.12), after 3 days for *P. putida* AF1I1 (27.2 ± 1.23 ppm) and for *B. ambifaria* AF8II10 (21.80 ± 0.29 ppm and 21.90 ± 0.45 ppm) on day 5 and day 7, respectively.

Plants exposed to stressful conditions produce excess ethylene which is detrimental to plant health. Plants develop many adaptive mechanisms such as ACC deaminase activity or to adapt to this new, hostile environment. The enzyme ACC deaminase (aminocyclopropane-1-carboxylic acid) produced by bacteria, enables the degradation of ACC, the main precursor of ethylene (Meena et al., 2020; Glick and Gamalero, 2021). In our study, all 33 selected isolates demonstrated ACC deaminase activity with three different levels. Almost 82% of the isolates showed moderate activity, whereas next 15% grew very rapidly on ACC as nitrogen source medium, indicating high ACC deaminase activity.

### Biocontrol potential of isolates

Germinating seeds are incessantly exposed to many hazardous factors, including pathogens, resulting in biotic stress (Brahim et al., 2022). The ability to produce siderophores and HCN was demonstrated by 18 and nine out of 33 isolates tested, respectively (Supplementary Table S3). Isolates tested exhibited an orange halo after a maximum of 10 days of incubation on a CAS agar plate were considered to be siderophore-producing bacteria. SPI was ranged from up to 6.17 (for *P. putida* AF1I1). Fourteen of the 18 isolates positives for siderophores production showed activity after only four days, while the other four isolates showed this ability only after 10 days only. For HCN production, seven isolates showed a very positive result and two demonstrated moderately positive result. Chitinolytic activity was detected in nine of 33 isolates tested with a range of CHI from 1.67 (*S. marcescens* AF8I6) to 2.5 (*S. plymuthica* EEPC5 and *B. toyonensis* EBV3), after 4 days of incubation. Clearing zones in CHDA medium gradually increased in most isolates. Maximum chitinolytic activity (CHI = 3.60) was shown by *B. toyonensis* EDC6 after 10 days of incubation. The high results obtained with isolates from the genus *Bacillus* are consistent with other studies (Drewnowska et al., 2020). In contrast to rhizospheric or soil isolates, the prevalence of chitinolytic activity was observed to be greater in endophytic isolates. The presence of these traits is extremely important as they have been shown to contribute to biocontrol and combat pathogenic microbes present in the environment. Encouragingly, nearly 40% of all strains exhibit at least two of the activities tested here, increasing their potential for biocontrol. The biocontrol properties tested are known to have broad activity against many pathogens (Shah et al., 2021). Therefore, selected strains can be successfully used as biocontrol agents, although none of the strains have all the tested properties simultaneously.

### Final selection of bacterial isolates for further studies

Based on the various above mentioned plant growth-promoting assays, 10 of the most potent bacterial isolates (Table 1) were further selected tested for compatibility, environmental adaptability by testing abiotic stress tolerance and determining the ability to use different carbon sources. The bacterial isolates were then tested for their efficiency in enhancing seed germination.

**Table 1:** Different plant growth-promoting traits of selected bacterial isolates after screening.

Isolates	PSI	KSI	IAA production (μg/mL)	ZSI			Ammonia production (μg/mL)	CHI	SPI	HCN production
	5 days	5 days	2 days	ZO	ZCHB	ZC	5 days	10 days	10 days	4 days
				4 days	4 days	4 days				
*Bacillus cereus* AF8II13	1.80	0.00	0.00	0.00	2.30	0.00	17.12 ± 1.19	1.75	2.00	−
*Bacillus toyonensis* EDC1	1.50	3.40	0.00	0.00	0.00	0.00	14.9 ± 1.14	2.70	0.00	−
*Burkholderia ambifaria* AF8II10	2.00	3.60	0.00	0.00	2.40	2.14	21.8 ± 0.29	0.00	1.33	−
*Klebsiella aerogenes* AF3II1	3.67	5.25	52.15	3.30	2.90	3.30	12.95 ± 0.45	0.00	0.00	−
*Pseudomonas putida* AF1I1	1.40	1.80	15.48	0.00	0.00	2.00	25.2 ± 1	0.00	6.00	++
*Pseudomonas fluorescens* AF8I4	3.00	1.83	2.21	0.00	1.80	1.90	15.21 ± 0.65	0.00	2.95	−
*Pseudomonas protegens* ML15	1.80	2.00	0.00	2.50	2.70	2.40	14.61 ± 1	0.00	1.45	+++
*Serratia marcescens* AF8I1	1.14	2.00	77.37	3.70	3.00	1.50	14.92 ± 0.63	2.70	1.25	−
*Serratia plymuthica* EDC15	1.40	0.00	119.19	0.00	2.10	0.00	10.96 ± 2.31	3.50	1.45	−
*Serratia plymuthica* EEPC5	1.50	3.09	119.07	0.00	2.40	1.50	13.9 ± 1.13	2.70	1.55	−

PSI, Phosphate solubilization index; KSI, Potassium solubilization index; IAA, Indole-3-acetic acid production; ZSI, zinc solubilization index. Zinc source was used separately as ZO, zinc oxide (ZnO, 0.15%); ZCHB, zinc carbonate hydroxide basic (2ZnCO_3_ × 3Zn(OH)_2_ 0.15%) and ZC, zinc carbonate (ZnCO_3_ 0.1%); CHI, chitinolytic index; SPI, siderophores production index; HCN, Hydrogen cyanide production; Key: + = positive, ++ = moderately positive, +++ = strongly positive, − = negative. All values given in the column are the average of three replicates.

#### Compatibility between isolates

To avoid antagonistic interactions between isolates in future consortia formulation compatibility was tested by plating on solid culture media. There was no incompatibility between 10 selected isolates, except that *P. fluorescens* ML15 is not compatible with *B. cereus* AF8II13 and *B. ambifaria* AF8II10; and *S. marcescens* AF8I1 is not compatible with *B. ambifaria* AF8II10 (Table 2). Results clearly indicated that isolate *B. ambifaria* AF8II10 can produce antimicrobials against pathogenic bacteria, similar to other isolates previously tested (Mullins et al., 2019).

**Table 2:** Cultural compatibility test of selected PGPB.

	*Bacillus cereus* AF8II13	*Bacillus toyonensis* EDC1	*Burkholderia ambifaria* AF8II10	*Klebsiella aerogenes* AF3II1	*Pseudomonas putida* AF1I1	*Pseudomonas fluorescens* AF8I4	*Pseudomonas protegens* ML15	*Serratia marcescens* AF8I1	*Serratia plymuthica* EDC15	*Serratia plymuthica* EEPC5
*Bacillus cereus* AF8II13	+	+	+	+	+	+	−	+	+	+
*Bacillus toyonensis* EDC1	+	+	+	+	+	+	+	+	+	+
*Burkholderia ambifaria* AF8II10	+	+	+	+	+	+	−	−	+	+
*Klebsiella aerogenes* AF3II1	+	+	+	+	+	+	+	+	+	+
*Pseudomonas putida* AF1I1	+	+	+	+	+	+	+	+	+	+
*Pseudomonas fluorescens* AF8I4	+	+	+	+	+	+	+	+	+	+
*Pseudomonas protegens* ML15	−	+	−	+	+	+	+	+	+	+
*Serratia marcescens* AF8I1	+	+	−	+	+	+	+	+	+	+
*Serratia plymuthica* EDC15	+	+	+	+	+	+	+	+	+	+
*Serratia plymuthica* EEPC5	+	+	+	+	+	+	+	+	+	+

#### Biochemical characterization

The ability to utilize carbon sources was determined in 10 selected strains as a supplement to metabolic profiling. Out the 35 carbon sources, only two substrates (dextrose and mannose) were utilized by all isolates tested (Table 3). Citrate and galactose could be utilized by all isolates except *B. thuringiensis* EDC1 and *B. cereus* AF8II13. Only two isolates were capable of degrading lactose (*K. aerogenes* AF3II1 and *S. plymuthica* EEPC5), and melezitose (*S. plymuthica* EDC15, *S. plymuthica* EEPC5). Isolate *K. aerogenes* AF3II1 was the most metabolically flexible of the isolates tested, being able to utilize 28 of 35 substrates tested. *K. aerogenes* AF3II1 was the only isolate that could utilize arabitol, rhamnose, and D-arabinose. Moreover, *S. plymuthica* EDC15 and *S. plymuthica* EEPC5 showed the ability to utilize 24 compounds. In contrast, *P. putida* AF1I1 showed the ability to utilize only five substrates, namely xylose, dextrose, galactose, mannose, and citrate, In addition, 70 and 80% of the isolates were able to utilize maltose and fructose, respectively. The available carbon source is a primary factor for microbial survival. Isolates capable of degrading multiple carbon sources are more likely to survive, especially in a new, unfavorable environment.

**Table 3:** Biochemical characterization of selected bacterial isolates.

Carbon source	Bacterial isolates									
	*P. putida* AF1I1	*K. aerogenes* AF3II1	*S. marcescens* AF8I1	*P. fluorescens* AF8I4	*B. ambifaria* AF8II10	*B. cereus* AF8II13	*B. toyonensis* EDC1	*S. plymuthica* EDC15	*S. plymuthica* EEPC5	*P. protegens* ML15
Lactose	−	+	−	−	−	−	−	−	+	−
Xylose	+	+	+	+	−	−	−	+	+	+
Maltose	−	+	+	−	+	+	+	+	+	−
Fructose	−	+	+	−	+	+	+	+	+	+
Dextrose	+	+	+	+	+	+	+	+	+	+
Galactose	+	+	+	+	+	−	+	+	+	+
Raffinose	−	+	−	−	−	−	−	+	+	−
Trehalose	−	+	+	−	−	+	+	+	+	+
Melibiose	−	+	+	+	−	−	−	+	+	+
Sucrose	−	+	+	+	−	+	−	+	+	−
L-Arabinose	−	+	+	+	−	−	−	+	+	+
Mannose	+	+	+	+	+	+	+	+	+	+
Inulin	−	+	+	−	+	+	+	+	+	+
Sodium gluconate	−	+	+	−	−	+	−	+	+	−
Glycerol	−	+	+	−	−	+	−	+	+	−
Salicin	−	+	+	−	+	−	−	+	+	−
Dulcitol	−	−	−	−	−	−	−	−	−	−
Inositol	−	+	+	−	−	−	−	+	+	−
Sorbitol	−	+	+	−	−	−	−	+	+	−
Mannitol	−	+	+	−	+	−	−	+	+	−
Adonitol	−	+	+	−	−	−	−	−	−	−
Arabitol	−	+	−	−	−	−	−	−	−	−
Erythritol	−	−	−	−	−	−	−	−	−	−
α-Methyl-D-glucoside	−	−	−	−	−	−	−	−	−	−
Rhamnose	−	+	−	−	−	−	−	−	−	−
Cellobiose	−	+	−	−	−	−	−	+	+	−
Melezitose	−	−	−	−	−	−	−	+	+	−
α-Methyl-D-mannoside	−	−	−	−	−	−	−	−	−	−
Xylitol	−	−	−	−	−	−	−	−	−	−
ONPG	−	+	−	−	−	−	−	+	+	−
Esculin hydrolysis	−	+	+	+	+	−	+	+	+	+
D-arabinose	−	+	−	−	−	−	−	+	−	−
Citrate	+	+	+	+	+	+	−	+	+	+
Malonate	−	+	−	+	+	−	−	−	−	+
Sorbose	−	−	−	−	−	−	−	−	−	−

Data based on three replicates. Key: + = positive, − = negative.

#### Abiotic stress tolerance

As previously indicated, microorganisms with a broad spectrum of activity and high tolerance to various stress conditions are needed. Abiotic stress affects both bacteria growth and plant development (Mahdi et al., 2020; Zhang et al., 2022). Therefore, isolates were cultured on different media to determine their potential to survive under various abiotic stresses. It was found that most of tested bacteria could tolerate salinity stress up to 6% NaCl in media (Table 4). Only isolate *B. ambifaria* AF8II1 showed the ability to grow in media enriched up to 10% NaCl. Drought stress was tolerated by all isolates when the potential reached −0.75 MPa. Further decrease in water potential clearly weakened the tolerance and survival of the tested bacteria. However, further analysis under lower potential showed that following exophytic isolates: *P. putida* AF1I1, *P. fluorescens* AF8I4, *B. ambifaria* AF8II10, *B. cereus* AF8II13, and *P. fluorescens* ML15 were able to grow under-1.2 MPa conditions, which can be hypothesized as due to continuous exposure of abiotic stresses, exophytes are more tolerant to it as compare to endophytes. Growth observation on LB media confirmed a wide range of pH tolerance. Among the different pH values tested, all the isolates showed maximum growth at pH 6 to 10, while the minimum growth of most of the bacterial isolates was observed at pH 4.

**Table 4:** Abiotic stresses tolerance of selected potent PGPB isolates.

Isolates	Abiotic stresses													
	Salinity						pH					Drought		
	2%	4%	6%	8%	10%	12%	4	5	6	8	10	−0.35 MPa	−0.75 MPa	−1.2 MPa
*P. putida* AF1I1														
*K. aerogenes* AF3II1														
*S. marcescens* AF8I1														
*P. fluorescens* AF8I4														
*B. ambifaria* AF8II10														
*B. cereus* AF8II13														
*B. toyonensis* EDC1														
*S. plymuthica* EDC15														
*S. plymuthica* EEPC5														
*P. protegens* ML15														

Data based on three replicates. Key: 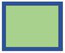
Positive 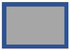
Negative.

### Carrot seed germination enhancement potential in bacteria

**Figure 3:**
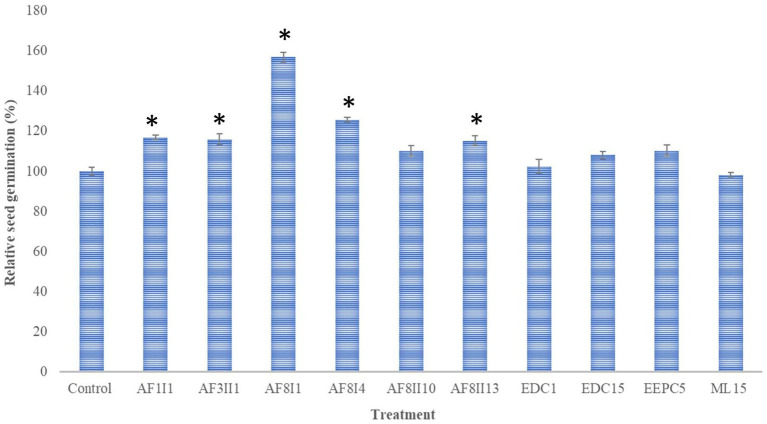
Effect of inoculation of 10 selected isolates on seed germination of *Daucus carota*. Bars representing mean values and standard errors and marked with * indicate statistically significant (*p* ≤ 0.05) differences from control. AF1I1, *P. putida* AF1I1; AF3II1, *K. aerogenes* AF3II1; AF8I1, *S. marcescens* AF8I1, AF8I4, *P. fluorescens* AF8I4; AF8II10, *B. ambifaria* AF8II10; AF8II13, *B. cereus* AF8II13; EDC1, *B. thuringiensis* EDC1; EDC15, *S. plymuthica* EDC15; EEPC5, *S. plymuthica* EEPC5; ML15, *P. fluorescens* ML15.

Seed germination of any plant requires optimal temperature and humidity to break exogenous dormancy. Some factors, such as hormones, can affect the rate and uniformity of mature seed germination (Begum et al., 2022). Carrot seeds, like other plants, do not germinate completely, but only 50–85%. From an economic point of view, the loss of seeds is high. Moreover, increasing the germination rate leads to higher yield and lower seed loss (Makhaye et al., 2021). In this study, the ability of 10 selected isolates to promote germination of carrot seed was investigated to select isolates with biostimulatory potential. Soaked carrot seeds were placed on Whatman paper drenched with the same bacterial suspension. The addition of the bacterial suspension was to support the colonization process and increase the number of cells adhering to the seeds. The potential to enhance seed germination was variable among the tested bacteria (Figure 3). Compared to control, *S. marcescens* AF8I1 significantly improved the relative seed germination by 156.88 ± 2.35%. In addition, carrot seeds inoculated with *P. fluorescens* AF8I4, *P. putida* AF1I1, *K. aerogenes* AF3II1, and *B. cereus* AF8II13 increased relative seed germination by 125.64 ± 12.1%, 116.8 ± 1.11%, 115.9 ± 2.67%, and 115.28 ± 2.56%, respectively. Furthermore, the results obtained for *P. putida* AF1I1 and *K. aerogenes* AF3II1 were not significantly different from each other, as were the results of treatment of *B. cereus* AF8II13 and *P. fluorescens* AF8I4. Similar to these studies, Rudolph et al. (2015) also showed that the use of strains identified as *P. fluorescens* and *P. putida* species can improve wheat seed germination.

Bacterial properties are well described in terms of plant growth promotion and biocontrol activity, but the importance of these attributes in enhancing seed germination is still poorly understood (Hagaggi and Mohamed, 2020; Mahdi et al., 2020; Pranaw et al., 2020). In this study, seeds responded differently to tested isolates, likely due to the individual properties and competencies of the isolates used (Majeed et al., 2015). The isolates *S. marcescens* AF8I1, *P. putida* AF1I1, *K. aerogenes* AF3II1, *P. fluorescens* AF8I4, and *B. cereus* AF8II13 raised seed germination between 15.28 and 56.88%. All of these isolates showed IAA production as well as phosphate and potassium solubilization ability, except for isolate *B. cereus* AF8II13, which in contrast showed only phosphate solubilization. The best result in this experiment was obtained with *P. fluorescens* AF8I1, which previously showed the ability to solubilize phosphate and a high activity in solubilizing potassium. In addition, this isolate showed ACC deaminase activity and strong IAA hormone synthesis. Several studies have shown that seed soaking by some IAA producing bacteria has a positive effect on seed germination. Maize seeds soaked in a bacterial suspension of the IAA producer *Mixta theicola* SAR resulted in a 27% improvement in germination (Hagaggi and Mohamed, 2020). IAA production among these strains was highly variable, i.e., *S. marcescens* AF8I1 showed high IAA production, while *B. cereus* AF8II13 did not synthesize this hormone. The remaining isolates showed IAA production ranging from 15.23 to 51.27 μg mL^−1^. It is worth mentioning that three of the isolates used *S. plymuthica* EDC15, *B. ambifaria* AF8II10, and *S. plymuthica* EEPC5 showed a positive effect on seed germination, i.e., they increased germination by 8.22, 10.22, and 10.32%, respectively. All these isolates showed the ability to produce auxins. However, the result was not statistically significant. In studies by Mahdi et al. (2020) the seed germination rate of quinoa (*Chenopodium quinoa*) was increased by 116 and 132% after the application of *Bacillus licheniformis* QA1 and *Enterobacter asburiae* QF11, respectively. These strains showed high phosphorus solubilization activity and QF11 exhibited overproduction of auxin and QA1 of ammonia and siderophores. Inoculation of tomato seeds with *Pseudomonas* sp. S3 resulted in a relative germination of 111% compared to the control. This strain was positive for the production of IAA, ACC deaminase, and siderophores and was a phosphate and zinc solubilizer (Pandey and Gupta, 2020). Brahim et al. (2022) demonstrated that *Bacillus pumilus* MA9 and *Virgibacillus halodenitrificans* MA14 significantly improved wheat germination rate due to secretion of auxin, solubilization of inorganic phosphate, and ACC deaminase activity. Comparison of these reports with the present data suggests that bacterial processes such as phosphate solubilization and ACC deaminase activity may also be involved in the induction of carrot seed germination.

However, it should be noted that various studies show that only certain strains are able to promote seed germination. For this reason, many attempts have failed. Treatment of *Cuscuta campestris* seeds with *Bacillus* sp. had no significant effect on seed germination compared to the control (Saric-Krsmanovic et al., 2017). Although there is evidence that isolates of the genus *Pseudomonas* sp. can produce hydrocyanic acid gas, *P. putida* AFI1 used in the experiment did not inhibit seed germination even though it produced HCN (Makhaye et al., 2021). The effect of HCN was demonstrated with isolate ML15, which was could produce HCN, but was unable to synthesis auxins. In addition, it is worth noting that all strains used showed the ability to solubilize Zn from at least one of the sources tested. Unfortunately, there are no data linking this bacterial activity to seed germination. Isolates that showed a positive effect on seed germination were characterized by different biocontrolling properties. Most of them showed only one of the investigated properties. The exception was isolate AF8II13, which showed both chitinolytic activity and the ability to produce siderophores, and AF1I1, which showed strong production of siderophores and HCN. Data from other studies mention the occurrence of features such as siderophores production in isolates that affect seed germination, and there is no evidence of these traits for this process (Pandey and Gupta, 2020). Findings suggest that there is no relationship between the presence of biocontrol abilities and the effect on seed germination. Siderophores chelate iron this help in iron acquisition by the plants, hence the production of these compounds can be extremely helpful in the acquisition of this element by developing plant.

## Conclusion

In conclusion, the application of some bacteria with plant growth-promoting traits can improve the germination of carrots. The results of this study show that mainly auxins are involved in seed germination. The ability of phosphate solubilization may play a role in seed germination, as shown by the positive effect of isolate AF8II13 on carrot seed germination in the absence of IAA synthesis. Since auxins can promote plant growth, these results indicate the great potential of some isolates as plant growth-promoting rhizobacteria, and as biostimulants for seed biopriming. It can also be suggested that HCN produced by bacteria does not have a decisive effect on seed germination. The evidence of biocontrolling activity seems to be insignificant in relation to seed germination, which does not exclude the great importance of these properties in protecting seeds and later the growing plant from pathogens.

## Data availability statement

The datasets presented in this study can be found in online repositories. The names of the repository/repositories and accession number(s) can be found in the article/Supplementary material.

## Author contributions

KP and AF: conceptualization. AF and NA: investigation and formal analysis. AF: writing – original draft preparation. AF, KP, and LD: writing – review and editing. KP: supervision. All authors have read and agreed to the published version of the manuscript.

## Funding

This research was funded by the grant from the project “The Fly ash as the precursors of functionalized materials for applications in environmental engineering, civil engineering and agriculture” (no. POIR.04.04.00-00-14E6/18-00), carried out within the TEAM-NET program of the Foundation for Polish Science co-financed by the European Union under the European Regional Development Fund.

## Conflict of interest

The authors declare that the research was conducted in the absence of any commercial or financial relationships that could be construed as a potential conflict of interest.

## Publisher’s note

All claims expressed in this article are solely those of the authors and do not necessarily represent those of their affiliated organizations, or those of the publisher, the editors and the reviewers. Any product that may be evaluated in this article, or claim that may be made by its manufacturer, is not guaranteed or endorsed by the publisher.

## Footnotes

^1^ http://www.nvbi.nlm.nih.gov
