# Supplementary material for: Biopriming of seed with plant growth-promoting bacteria for improved germination and seedling growth
Source: Front Microbiol. 2023 Feb 28;14:1142966. doi: 10.3389/fmicb.2023.1142966 (PMC10011460; doi:10.3389/fmicb.2023.1142966)
Supplement: Supplementary file 1 [file Table_1.DOCX]

Supplementary Material

**Table S1.** Isolated bacterial strains with their origin and preliminary results of phosphate and potassium solubilization and IAA synthesis ability.

| **Numbers** | **Isolate^1^** | **Origin^2^**  (Place, crop, sample type) | **PSI^3^** | **KSI^4^** | **IAA. µg/ml^5^** | **IAA. µg/ml^6^** |
| --- | --- | --- | --- | --- | --- | --- |
|  |  |  | 5 days | 5 days | 48h | 5 days |
| 1 | AF1I1 | Hermanówka, rye, rhizopheric soil | 1.40 | 1.80 | 15.48 | 15.86 |
| 2 | AF1I2 |  | 2.00 | 1.50 | 0.00 | 0.00 |
| 3 | AF1I4 |  | 1.41 | 0.00 | 0.00 | 0.00 |
| 4 | AF1I6 |  | 1.67 | 0.00 | 0.00 | 0.00 |
| 5 | AF1I7 |  | 1.80 | 1.36 | 0.00 | 0.00 |
| 6 | AF1I8 |  | 3.00 | 1.25 | 78.59 | 67.76 |
| 7 | AF1I9 |  | 1.20 | 1.29 | 88.35 | 117.41 |
| 8 | AF2I2 | Niewodnica Nargilewska, rye, rhizopheric soil | 1.25 | 0.00 | 52.24 | 35.76 |
| 9 | AF2I3 |  | 0.00 | 0.00 | 81.41 | 47.76 |
| 10 | AF2I4 |  | 1.44 | 1.36 | 61.65 | 65.65 |
| 11 | AF2I5 |  | 1.38 | 0.00 | 53.18 | 25.41 |
| 12 | AF2I5A |  | 0.00 | 0.00 | 78.12 | 39.29 |
| 13 | AF2I7 |  | 2.50 | 3.57 | 35.41 | 39.53 |
| 14 | AF2I8 |  | 1.50 | 1.30 | 37.88 | 10.59 |
| 15 | AF2II2 |  | 0.00 | 3.67 | 14.82 | 2.59 |
| 16 | AF2II3 |  | 1.25 | 1.14 | 99.41 | 140.00 |
| 17 | AF2II6 |  | 1.50 | 1.25 | 20.47 | 27.53 |
| 18 | AF3I1 |  | 1.33 | 0.00 | 30.59 | 123.53 |
| 19 | AF3I2 |  | 0.00 | 0.00 | 0.00 | 70.82 |
| 20 | AF3I3 |  | 1.75 | 1.40 | 44.47 | 38.12 |
| 21 | AF3I4 |  | 2.00 | 1.29 | 32.35 | 24.00 |
| 22 | AF3I5 |  | 2.00 | 1.86 | 48.71 | 106.82 |
| 23 | AF3II1 |  | 3.67 | 5.25 | 52.15 | 51.27 |
| 24 | AF3II2 |  | 1.20 | 0.00 | 43.88 | 102.82 |
| 25 | AF3II4 |  | 1.11 | 1.55 | 33.18 | 17.41 |
| 26 | AF3II5 |  | 1.20 | 1.30 | 40.94 | 78.12 |
| 27 | AF3II6 |  | 1.20 | 1.25 | 28.24 | 52.24 |
| 28 | AF3II7 |  | 1.20 | 1.30 | 25.41 | 64.47 |
| 29 | AF3II8 |  | 2.50 | 2.00 | 76.59 | 46.12 |
| 30 | AF3II9 |  | 2.25 | 2.33 | 9.41 | 22.35 |
| 31 | AF3II10 |  | 0.00 | 0.00 | 79.88 | 30.12 |
| 32 | AF3II11 |  | 1.43 | 1.33 | 19.76 | 30.12 |
| 33 | AF4I2 | Rumejki, maize, rhizopheric soil | 1.50 | 1.20 | 64.00 | 92.47 |
| 34 | AF4I4 |  | 1.29 | 2.00 | 95.53 | 118.12 |
| 35 | AF4I6 |  | 1.33 | 0.00 | 63.28 | 71.33 |
| 36 | AF4II1 |  | 1.47 | 1.60 | 0.00 | 0.00 |
| 37 | AF4II1A |  | 1.10 | 0.00 | 49.65 | 0.00 |
| 38 | AF4II2 |  | 1.50 | 3.40 | 72.00 | 104.47 |
| 39 | AF4II3 |  | 1.25 | 2.17 | 0.00 | 0.00 |
| 40 | AF4II4 |  | 1.50 | 0.00 | 92.12 | 96.94 |
| 41 | AF4II5 |  | 1.29 | 0.00 | 171.18 | 122.59 |
| 42 | AF5I3 |  | 0.00 | 2.40 | 0.00 | 100.47 |
| 43 | AF5I6 |  | 1.20 | 1.80 | 0.00 | 0.00 |
| 44 | AF5I9 |  | 1.80 | 1.20 | 0.00 | 79.06 |
| 45 | AF5I10 |  | 1.33 | 0.00 | 80.24 | 87.76 |
| 46 | AF5I11 |  | 1.33 | 2.67 | 96.35 | 79.53 |
| 47 | AF5I12 |  | 1.56 | 1.80 | 11.65 | 16.71 |
| 48 | AF6I1 | Hołodolina, triticale, rhizopheric soil | 1.20 | 3.20 | 0.00 | 24.24 |
| 49 | AF6I3 |  | 1.10 | 0.00 | 77.06 | 63.53 |
| 50 | AF6I4 |  | 2.25 | 0.00 | 0.00 | 53.88 |
| 51 | AF6I5 |  | 0.00 | 0.00 | 131.06 | 215.29 |
| 52 | AF6I6 |  | 1.20 | 1.29 | 130.82 | 202.82 |
| 53 | AF6I8 |  | 1.20 | 1.00 | 134.12 | 180.47 |
| 54 | AF7I2 | Hołodolina, maize, rhizopheric soil | 1.38 | 1.30 | 123.29 | 210.12 |
| 55 | AF7I3 |  | 0.00 | 0.00 | 0.00 | 97.88 |
| 56 | AF7I6 |  | 0.00 | 0.00 | 0.00 | 0.00 |
| 57 | AF7I7 |  | 0.00 | 2.58 | 14.00 | 0.00 |
| 58 | AF7IB |  | 1.50 | 1.13 | 6.47 | 44.00 |
| 59 | AF7I11 |  | 0.00 | 0.00 | 0.00 | 71.53 |
| 60 | AF7I13 |  | 1.33 | 0.00 | 56.12 | 35.06 |
| 61 | AF7II2 |  | 1.10 | 0.00 | 0.00 | 52.71 |
| 62 | AF7II3 |  | 1.25 | 2.67 | 147.18 | 118.12 |
| 63 | AF8I1 |  | 1.14 | 2.00 | 77.37 | 38.38 |
| 64 | AF8I2 |  | 2.43 | 1.40 | 0.00 | 19.76 |
| 65 | AF8I4 |  | 3.00 | 1.83 | 2.21 | 15.23 |
| 66 | AF8I6 |  | 1.60 | 3.00 | 89.95 | 65.30 |
| 67 | AF8II1 |  | 0.00 | 0.00 | 92.82 | 49.41 |
| 68 | AF8II2 |  | 1.29 | 0.00 | 92.00 | 52.71 |
| 69 | AF8II3 |  | 1.20 | 0.00 | 0.00 | 0.00 |
| 70 | AF8II6 |  | 0.00 | 0.00 | 0.00 | 0.00 |
| 71 | AF8II7 |  | 2.00 | 2.00 | 0.00 | 0.00 |
| 72 | AF8II10 |  | 2.00 | 3.60 | 0.00 | 0.00 |
| 73 | AF8II12 |  | 0.00 | 1.17 | 88.82 | 57.88 |
| 74 | AF8II13 |  | 1.80 | 0.00 | 0.00 | 0.00 |
| 75 | AF8II14 |  | 2.30 | 1.25 | 2.72 | 14.86 |
| 76 | AF8II15 |  | 0.00 | 0.00 | 73.53 | 36.71 |
| 77 | AF9I1 | Hołodolina, oat, rhizopheric soil | 1.78 | 1.45 | 38.00 | 87.29 |
| 78 | AF9I2 |  | 1.10 | 0.00 | 0.00 | 15.06 |
| 79 | AF9I3 |  | 1.25 | 1.43 | 51.29 | 39.29 |
| 80 | AF9II1 |  | 0.00 | 0.00 | 93.29 | 44.94 |
| 81 | AF9II1 |  | 0.00 | 0.00 | 0.12 | 0.00 |
| 82 | AF9II2 |  | 0.00 | 0.00 | 11.76 | 38.59 |
| 83 | AF10I1 | Hołodolina, rye, rhizopheric soil | 1.78 | 0.00 | 0.20 | 0.00 |
| 84 | AF10I2 |  | 1.40 | 2.00 | 88.47 | 56.94 |
| 85 | AF10I3 |  | 0.00 | 0.00 | 0.00 | 100.71 |
| 86 | AF10I4 |  | 0.00 | 0.00 | 0.00 | 71.06 |
| 87 | AF10I6 |  | 1.10 | 1.25 | 0.00 | 18.35 |
| 88 | BV2-1 | Białystok, Beet, rhizospheric soil | 1.30 | 2.80 | 133.53 | 167.53 |
| 89 | BV2-2 |  | 2.00 | 2.00 | 8.24 | 56.24 |
| 90 | BV2-3 |  | 0.00 | 0.00 | 96.82 | 202.35 |
| 91 | BV2-4 |  | 0.00 | 0.00 | 0.00 | 0.00 |
| 92 | BV2-5 |  | 0.00 | 0.00 | 0.20 | 0.26 |
| 93 | DC1 | Białystok, Carrot, rhizospheric soil | 1.30 | 2.00 | 20.94 | 89.41 |
| 94 | DC1A |  | 0.00 | 0.00 | 35.29 | 48.94 |
| 95 | DC2 |  | 0.00 | 0.00 | 80.82 | 24.00 |
| 96 | DC3 |  | 1.20 | 0.00 | 11.41 | 17.18 |
| 97 | DC4 |  | 2.00 | 1.56 | 0.00 | 0.00 |
| 98 | DC5 |  | 2.00 | 3.25 | 0.00 | 0.00 |
| 99 | DC6 |  | 1.60 | 1.60 | 52.82 | 91.29 |
| 100 | DC7 |  | 0.00 | 2.20 | 34.24 | 60.94 |
| 101 | DC8 |  | 1.10 | 1.43 | 42.12 | 56.71 |
| 102 | DC9 |  | 1.40 | 1.40 | 81.71 | 66.24 |
| 103 | DC10 |  | 1.10 | 1.23 | 20.94 | 17.88 |
| 104 | DC11 |  | 1.40 | 1.13 | 41.29 | 105.41 |
| 105 | DC12 |  | 1.30 | 1.17 | 39.06 | 89.65 |
| 106 | DC13 |  | 1.10 | 0.00 | 0.00 | 100.94 |
| 107 | DC14 |  | 1.30 | 1.45 | 15.88 | 19.53 |
| 108 | DC16 |  | 1.10 | 2.00 | 49.53 | 93.18 |
| 109 | DC2-1 |  | 0.00 | 0.00 | 138.82 | 113.65 |
| 110 | DC2-2 |  | 1.10 | 0.00 | 45.65 | 64.24 |
| 111 | DC2-3 |  | 0.00 | 0.00 | 123.41 | 152.94 |
| 112 | DC2-4 |  | 1.10 | 1.25 | 18.82 | 37.88 |
| 113 | DC2-5 |  | 0.00 | 0.00 | 18.12 | 97.88 |
| 114 | EBV1 | Białystok, Beet, endophytic tissues | 1.80 | 1.50 | 2.09 | 0.00 |
| 115 | EBV2 |  | 0.00 | 0.00 | 67.29 | 23.29 |
| 116 | EBV3 |  | 0.00 | 1.43 | 0.00 | 0.00 |
| 117 | EBV4 |  | 1.10 | 1.57 | 9.65 | 44.47 |
| 118 | EBV5 |  | 1.50 | 1.21 | 80.94 | 52.47 |
| 119 | EBV6 | Białystok, Beet, endophytic tissues | 0.00 | 0.00 | 0.00 | 0.00 |
| 120 | EBV7 |  | 0.00 | 0.00 | 38.12 | 74.59 |
| 121 | EBV8 |  | 1.20 | 1.63 | 66.59 | 53.88 |
| 122 | EBV10 |  | 1.30 | 2.00 | 1.41 | 73.41 |
| 123 | EBV11 |  | 0.00 | 2.14 | 20.59 | 36.94 |
| 124 | EBV12 |  | 0.00 | 2.83 | 0.00 | 0.00 |
| 125 | EBV2-1 |  | 1.50 | 0.00 | 79.29 | 53.41 |
| 126 | EBV2-2 |  | 0.00 | 2.60 | 42.47 | 72.47 |
| 127 | EBV2-3 |  | 0.00 | 0.00 | 0.00 | 69.41 |
| 128 | EBV2-4 |  | 1.50 | 2.00 | 0.00 | 0.00 |
| 129 | EBV2-5 |  | 2.00 | 2.00 | 5.11 | 6.62 |
| 130 | EBV2-6 |  | 0.00 | 1.33 | 0.00 | 49.41 |
| 131 | EBV2-7 |  | 0.00 | 0.00 | 67.76 | 89.88 |
| 132 | EBV2-9 |  | 0.00 | 0.00 | 60.59 | 63.53 |
| 133 | EBV2-10 |  | 0.00 | 0.00 | 0.00 | 0.00 |
| 134 | EDC1 | Białystok, Carrot, endophytic tissues | 1.50 | 3.40 | 0.00 | 0.00 |
| 135 | EDC5 |  | 1.40 | 2.60 | 0.00 | 0.00 |
| 136 | EDC6 |  | 0.00 | 0.00 | 41.08 | 93.41 |
| 137 | EDC8 |  | 0.00 | 0.00 | 82.35 | 41.18 |
| 138 | EDC9 |  | 1.20 | 2.17 | 96.62 | 92.91 |
| 139 | EDC15 |  | 1.40 | 0.00 | 119.19 | 102.03 |
| 140 | EEPC1 |  | 0.00 | 2.00 | 114.71 | 132.24 |
| 141 | EEPC2 |  | 0.00 | 0.00 | 0.00 | 23.29 |
| 142 | EEPC5 |  | 1.50 | 3.09 | 119.07 | 84.54 |
| 143 | AFI | Israel, Dead Sea mud from the shore | 0.00 | 2.50 | 2.65 | 17.43 |
| 144 | AFII1 |  | 0.00 | 0.00 | 1.84 | 3.53 |
| 145 | AFII3 |  | 1.33 | 0.00 | 1.40 | 3.22 |
| 146 | ML7 | Błędowska Desert, Sand | 1.20 | 0.00 | 0.00 | 0.00 |
| 147 | ML8 |  | 2.40 | 2.00 | 0.00 | 0.00 |
| 148 | ML12 |  | 1.60 | 0.00 | 3.28 | 0.00 |
| 149 | ML14 |  | 1.40 | 2.00 | 0.00 | 0.00 |
| 150 | ML15 |  | 1.80 | 2.00 | 0.00 | 0.00 |
| 151 | PC1 | Białystok, Parsley, rhizospheric soil | 1.30 | 3.25 | 16.47 | 0.00 |
| 152 | PC2 |  | 1.50 | 0.00 | 11.65 | 25.65 |
| 153 | PC2 |  | 1.50 | 0.00 | 0.00 | 1.65 |
| 154 | PC3 |  | 1.50 | 2.86 | 144.24 | 65.88 |
| 155 | PC4 |  | 0.00 | 1.13 | 5.29 | 23.76 |
| 156 | PC5 |  | 1.20 | 3.60 | 14.59 | 1.18 |

^1^ Code of newly isolated bacterial isolate.
^2^ Origin of sample (place, crop and sample type)
^3^ PSI, phosphate solubilization index determined by calculating using the following formula: (diameter of the clearing zone + colony diameter)/colony diameter.
^4^ KSI, potassium solubilization index determined by calculating using the following formula: (diameter of the clearing zone + colony diameter)/colony diameter.
^5^ Indole-3-acetic acid (IAA) production results after 48 h of incubation.
^6^ IAA production results after 5 days of incubation.

**Table S2.** Identification of 33 selected potential plant growth promoting bacteria.

| **Strain^1^** | **16S rRNA Gene length (bp)** | **Accession number for the 16S rRNA Gene Sequence^2^** | **Closely Related Species^3^** | **Gene bank ID of related strain^3^** | **Accession number of the 16S rRNA Gene sequence of related strain^3^** | **Identity (%)** |
| --- | --- | --- | --- | --- | --- | --- |
| **AF1I1** | 1486 | OM250436 | *Pseudomonas putida* | 32zhy | AM411059.1 | 100.00 |
| **AF1I7** | 1486 | OM250438 | *Pseudomonas koreensis* | AC5.1 | EU275363.1 | 100.00 |
| **AF3II1** | 1487 | OM250432 | *Klebsiella aerogenes* | 18-2341 | CP049600.1 | 99.87 |
| **AF4I6** | 1486 | OM250459 | *Pseudomonas fluorescens* | 90F12-2 | KT695840.1 | 100.00 |
| **AF8I1** | 1491 | OM250451 | *Serratia marcescens* | N10A28 | CP033623.1 | 99.87 |
| **AF8I4** | 1486 | OM250437 | *Pseudomonas fluorescens* | L111 | CP015638.1 | 100.00 |
| **AF8I6** | 1491 | OM250450 | *Serratia marcescens* | BWH-23 | CP020501.1 | 99.80 |
| **AF10I1** | 1485 | OM250440 | *Pseudomonas azotoformans* | P45A | CP041236.1 | 99.87 |
| **AF8II7** | 1482 | OM250463 | *Burkholderia ambifaria* | FDAARGOS_1027 | CP066037.1 | 100.00 |
| **AF8II10** | 1482 | OM250462 | *Burkholderia ambifaria* | FDAARGOS_1027 | CP066037.1 | 99.80 |
| **AF8II13** | 1500 | OM250442 | *Bacillus cereus* | FDAARGOS_918 | CP065650.1 | 99.87 |
| **AF8II14** | 1486 | OM250458 | *Pseudomonas fluorescens* | L111 | CP015638.1 | 100.00 |
| **BV2-5** | 1501 | OM250461 | *Lysinibacillus sphaericus* | DSM 28 | CP019980.1 | 99.93 |
| **DC5** | 1478 | OM250441 | *Comamonas koreensis* | T50-37 | CP043575.1 | 100.00 |
| **DC9** | 1433 | OM250456 | *Agrobacterium tumefaciens* | A6 | CP033028.1 | 99.93 |
| **EBV1** | 1501 | OM250447 | *Bacillus pseudomycoides* | BTZ | CP009651.1 | 100.00 |
| **EBV2-4** | 1485 | OM250439 | *Pseudomonas fluorescens* | 90F12-2 | KT695840.1 | 99.87 |
| **EBV2-5** | 1486 | OM250431 | *Pseudomonas brassicacearum* | Delaware | KT695846.1 | 100.00 |
| **EBV3** | 1501 | OM250445 | *Bacillus toyonensis* | SMP1.3ISP2 | MT052648.1 | 99.93 |
| **EBV12** | 1500 | OM250448 | *Brevibacterium frigoritolerans* | IHB B 15619 | MF800956.1 | 100.00 |
| **EDC1** | 1501 | OM250444 | *Bacillus toyonensis* | SMP1.3ISP2 | MT052648.1 | 100.00 |
| **EDC5** | 1486 | OM250460 | *Pseudomonas poae* | S09G 359 | CP025263.1 | 100.00 |
| **EDC6** | 1501 | OM250443 | *Bacillus toyonensis* | SMP1.3ISP2 | MT052648.1 | 100.00 |
| **EDC9** | 1433 | OM250457 | *Agrobacterium tumefaciens* | A7 | CP033028.1 | 99.93 |
| **EDC15** | 1491 | OM250453 | *Serratia plymuthica* | C-1 | CP053398.1 | 99.87 |
| **EEPC5** | 1490 | OM250452 | *Serratia plymuthica* | NCTC12961 | LS483469.1 | 99.80 |
| **AFI** | 1513 | OM250449 | *Oceanobacillus iheyensis* | CHQ24 | CP020357.1 | 99.80 |
| **AFII1** | 1502 | OM250455 | *Staphylococcus xylosus* | 2.1523 | CP066721.1 | 100.00 |
| **AFII3** | 1502 | OM250454 | *Staphylococcus xylosus* | 2.1523 | CP066721.1 | 100.00 |
| **ML8** | 1488 | OM250433 | *Pseudomonas protegens* | SN15-2 | CP043179.1 | 100.00 |
| **ML12** | 1501 | OM250446 | *Bacillus toyonensis* | SMP1.3ISP3 | MT052648.1 | 99.93 |
| **ML14** | 1488 | OM250434 | *Pseudomonas protegens* | SN 15-2 | CP043179.1 | 100.00 |
| **ML15** | 1488 | OM250435 | *Pseudomonas protegens* | SN 15-2 | CP043179.1 | 100.00 |
| ^1^ Name of the isolate according Table 1. ^2^ Accession numbers after isolated were deposited in the NCBI GenBank database. ^3^ The NCBI Genbank accession number of sequences, closely related species with the similarity. | | | | | | |

**Table S3.** Plant growth promoting traits of isolated bacteria after primary selection.

| **Isolate^1^** | **Ammonia production (ppm)** | | | | **CHI^2^** | | **ZSI^3^** | | | **SPI^6^** | | **HCN** |
| --- | --- | --- | --- | --- | --- | --- | --- | --- | --- | --- | --- | --- |
|  |  |  |  |  |  |  | **ZO** | **ZCHB** | **ZC** |  |  |  |
|  | **1 day** | **3 days** | **5 days** | **7 days** | **5 days** | **10 days** | **4 days** | | | **4 days** | **10 days** | **4 days** |
| AF1I1 | 11.40±0.23 | 27.2±1.23 | 25.2±1 | 21.2±0.99 | 0.00 | 0.00 | 0.00 | 0.00 | 2.00 | 6.15 | 6.00 | ++ |
| AF1I7 | 9.93±0.12 | 10.18±0.08 | 10.32±0.06 | 12.95±0.57 | 0.00 | 0.00 | 0.00 | 0.00 | 2.30 | 0.00 | 0.00 | ++ |
| AF3II1 | 10.93±0.01 | 11.48±1.08 | 12.95±0.45 | 8.77±0.13 | 0.00 | 0.00 | 3.30 | 2.90 | 3.30 | 0.00 | 0.00 | - |
| AF4I6 | 7.65±0.36 | 9.08±0.02 | 9.94±0.11 | 10.72±0.33 | 0.00 | 0.00 | 0.00 | 1.90 | 1.84 | 0.00 | 0.00 | - |
| AF8I1 | 8.25±0.18 | 9.35±0.29 | 14.92±0.63 | 13.61±1.81 | 2.30 | 2.70 | 3.70 | 3.00 | 1.50 | 1.15 | 1.25 | - |
| AF8I4 | 13.08±0.44 | 13.44±0.93 | 15.21±0.65 | 11.72±0.28 | 0.00 | 0.00 | 0.00 | 1.80 | 1.90 | 2.15 | 2.95 | - |
| AF8I6 | 8.22±0.13 | 8.26±0.08 | 16.89±0.65 | 12.99±0.11 | 1.67 | 2.00 | 3.70 | 3.20 | 0.00 | 1.05 | 1.19 | - |
| AF8II7 | 7.44±0.52 | 9.99±0.1 | 10.13±0.12 | 13.4±0.05 | 0.00 | 0.00 | 0.00 | 2.20 | 2.00 | 0.00 | 0.00 | - |
| AF10I1 | 6.20±0.12 | 8.5±0.13 | 4.9±0.12 | 9.8±0.05 | 0.00 | 0.00 | 0.00 | 1.60 | 1.47 | 1.20 | 1.25 | +++ |
| AF8II10 | 8.30±0.06 | 16.7±0.21 | 21.8±0.29 | 21.9±0.46 | 0.00 | 0.00 | 0.00 | 2.40 | 2.14 | 0.00 | 1.33 | - |
| AF8II13 | 4.30±0.27 | 12.94±0.31 | 17.12±1.19 | 15.58±1.13 | 1.80 | 1.75 | 0.00 | 2.30 | 0.00 | 1.90 | 2.00 | - |
| AF8II14 | 0.80±0.08 | 3.6±0.07 | 5±0.07 | 8.9±0.12 | 0.00 | 0.00 | 0.00 | 1.80 | 2.20 | 2.55 | 2.90 | - |
| BV2-5 | 9.28±0.13 | 9.92±0.22 | 15.21±0.23 | 16.57±0.34 | 0.00 | 0.00 | 0.00 | 0.00 | 0.00 | 0.00 | 0.00 | - |
| DC5 | 10.64±0.05 | 10.74±0.69 | 12.57±0.24 | 11.16±0.22 | 0.00 | 0.00 | 0.00 | 2.20 | 0.00 | 0.00 | 0.00 | - |
| DC9 | 10.6±0.05 | 13.1±0.09 | 12.5±0.1 | 14.7±0.35 | 0.00 | 0.00 | 0.00 | 3.50 | 2.20 | 1.45 | 1.25 | +++ |
| EBV1 | 4.6±0.02 | 4±0.06 | 7.8±0.07 | 10.7±0.04 | 0.00 | 0.00 | 0.00 | 1.60 | 0.00 | 0.00 | 0.00 | - |
| EBV2-4 | 9.9±0.04 | 15.3±0.06 | 12.4±0.07 | 16±0.12 | 0.00 | 0.00 | 0.00 | 0.00 | 2.80 | 1.15 | 1.80 | +++ |
| EBV2-5 | 8.97±0.12 | 14.05±0.06 | 18±0.07 | 20.86±0.32 | 0.00 | 0.00 | 0.00 | 0.00 | 1.80 | 1.80 | 1.70 | +++ |
| EBV3 | 4.75±0.08 | 14.61±0.82 | 16.4±1.3 | 13.97±0.17 | 2.50 | 2.30 | 0.00 | 1.90 | 1.60 | 0.00 | 0.00 |  |
| EBV12 | 0.34±0.05 | 0.88±0.11 | 1.5±0.14 | 1.8±0.12 | 0.00 | 0.00 | 0.00 | 0.00 | 0.00 | 0.00 | 0.00 | - |
| EDC1 | 7.39±0.60 | 10.4±0.72 | 14.9±1.14 | 13.42±0.36 | 2.50 | 2.70 | 0.00 | 0.00 | 0.00 | 0.00 | 0.00 | - |
| EDC5 | 13.9±0.23 | 11.9±0.29 | 11.6±0.29 | 13.6±0.13 | 0.00 | 0.00 | 0.00 | 4.00 | 2.05 | 1.15 | 1.95 |  |
| EDC6 | 5.47±0.05 | 6.72±0.08 | 13.59±0.1 | 13.03±0.1 | 2.30 | 3.60 | 0.00 | 0.00 | 2.30 | 0.00 | 0.00 | - |
| EDC9 | 12.30±0.22 | 14.4±0.19 | 12.3±0.17 | 15.7±0.16 | 0.00 | 0.00 | 0.00 | 1.60 | 1.70 | 0.00 | 0.00 | - |
| EDC15 | 6.62±0.16 | 6.89±0.57 | 10.96±2.31 | 7.24±1.3 | 2.45 | 3.50 | 0.00 | 2.10 | 0.00 | 1.35 | 1.45 | - |
| EEPC5 | 7.32±0.25 | 11.94±0.67 | 13.9±1.13 | 12.41±0.65 | 2.50 | 2.70 | 0.00 | 2.40 | 1.50 | 1.45 | 1.55 | - |
| AFI | 5.00±0.11 | 6.12±0.23 | 7.12±0.22 | 3.2±0.56 | 0.00 | 0.00 | 0.00 | 5.10 | 0.00 | 0.00 | 1.15 | - |
| AFII1 | 13.50±0.12 | 18.6±0.23 | 16.2±0.22 | 15.3±0.1 | 0.00 | 0.00 | 0.00 | 0.00 | 0.00 | 0.00 | 0.00 | - |
| AFII3 | 10.40±0.07 | 23.9±0.35 | 14.5±1.01 | 18.4±0.79 | 0.00 | 0.00 | 0.00 | 1.80 | 0.00 | 0.00 | 0.00 | - |
| ML8 | 3.4±0.22 | 7.7±0.13 | 5.9±0.18 | 6.6±0.12 | 0.00 | 0.00 | 2.13 | 2.50 | 1.80 | 1.40 | 1.45 | +++ |
| ML12 | 0.00 | 4.2±0.09 | 3.6±0.08 | 5.4±0.07 | 2.00 | 2.20 | 0.00 | 0.00 | 0.00 | 0.00 | 0.00 | - |
| ML14 | 9.31±0.23 | 10.15±0.1 | 11.74±0.09 | 13.87±0.62 | 0.00 | 0.00 | 2.80 | 2.90 | 1.50 | 0.00 | 1.50 | +++ |
| ML15 | 11.02±0.41 | 11.18±0.55 | 14.61±1 | 12.22±1.1 | 0.00 | 0.00 | 2.50 | 2.70 | 2.40 | 0.00 | 1.45 | +++ |

Values given in the column are the average of three replications. Key: + = positive, ++ = moderately positive, +++ = strongly positive, − = negative. ^1^ Name of newly isolated bacterial isolate identified according to Table S2.
^2^ CHI, chitinolytic index.
^3^ ZSI, zinc solubilization index. Zinc source was used separately as ZO, zinc oxide (ZnO, 0.15%); ZCHB, zinc carbonate hydroxide basic (2ZnCO_3_×3Zn(OH)_2_ 0.15%) and ZC, zinc carbonate (ZnCO_3_ 0.1%).
^6^ SPI, siderophores production index.
^7^ Inoculation time.
